# Supplementary material for: The controlled disassembly of mesostructured perovskites as an avenue to fabricating high performance nanohybrid catalysts
Source: Nat Commun. 2017 May 25;8:15553. doi: 10.1038/ncomms15553 (PMC5458515; doi:10.1038/ncomms15553)
Supplement: Supplementary Information — Supplementary Figures, Supplementary Methods, Supplementary Discussion, Supplementary Tables and Supplementary References [file ncomms15553-s1.pdf]

Supplementary Methods

PMMA template synthesis

Synthesis of the monodispersed poly(methyl methacrylate) PMMA template involves several steps as reported previously [1] and as shown in Supplementary Fig. 1. Firstly, 115 mL of methyl methacrylate was mixed with 1500 mL of water in a three-neck flask and stirred at 70 °C for 0.5 h while degassing with flowing N_2. Next, 0.4 g potassium persulfate ($K_2S_2O_8$) dissolved in 50 mL deionized water was rapidly added to the flask with the resulting suspension stirred at 70 °C for 2h. The PMMA microsphere suspension was centrifuged at 10000 rpm for 30 min and then re-dispersed in 3000 mL water. This step is designed to ensure the removal of K and S ions. Under the force of gravity, the well-ordered colloidal crystal templates were evaporated at 70 °C using a water bath. After drying overnight at room temperature (RT), the obtained PMMA microspheres were ready to be used as a template.

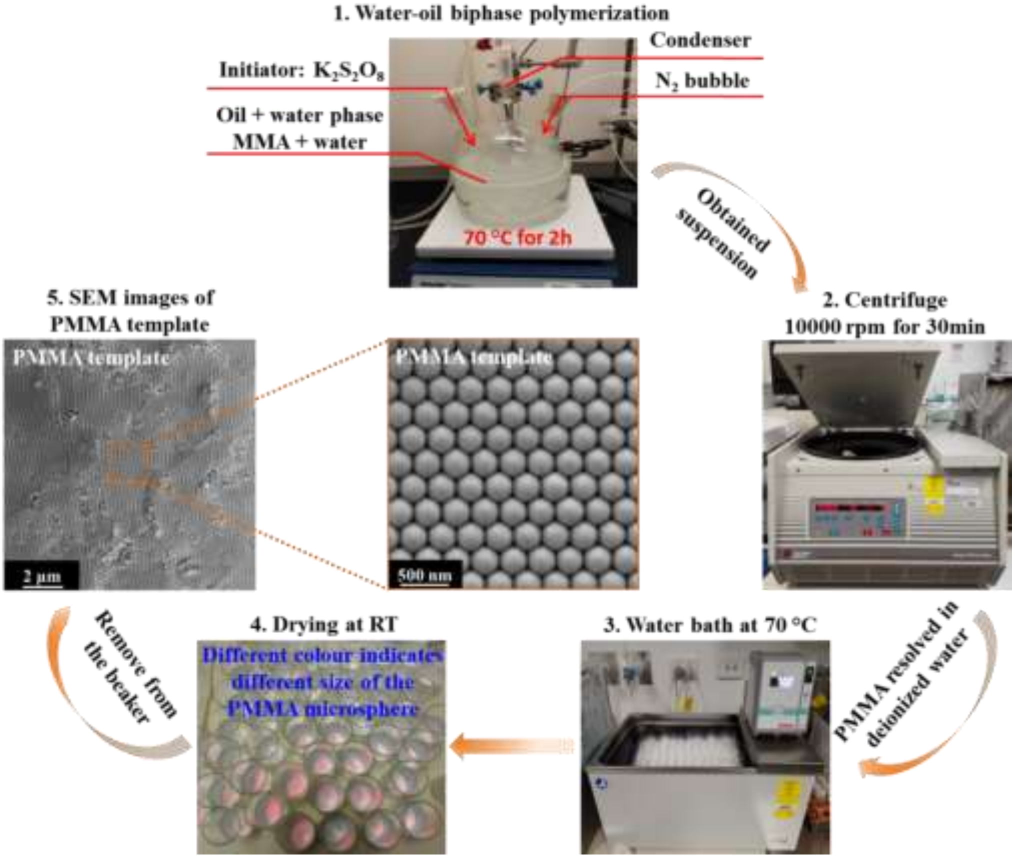

Supplementary Figure 1 Equipment and process used to synthesize PMMA microspheres.

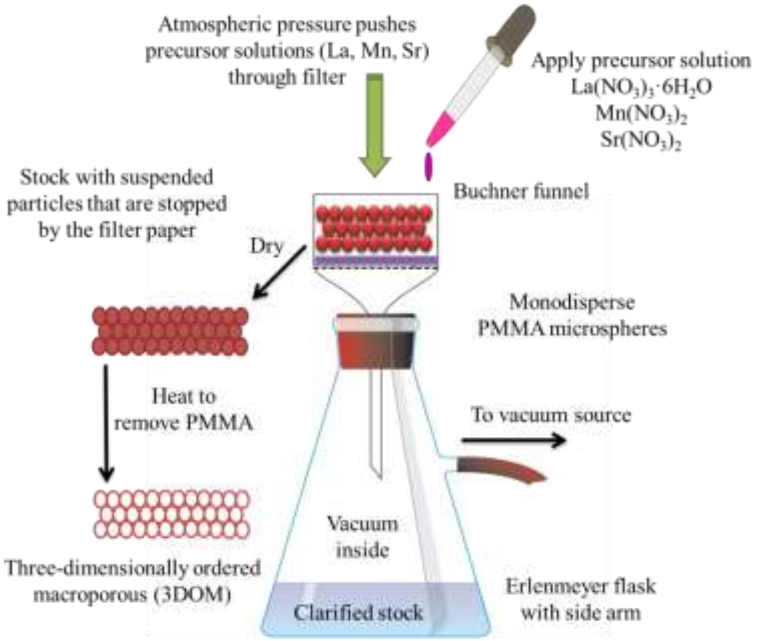

Supplementary Figure 2 Equipment and process used to synthesize 3DOM LSMO.

3D hexapod mesostructured LSMO perovskite synthesis

Three-dimensional hexapod mesostructured (3D-hm) LSMO was synthesized by a sonication method using ultrasound as external force to cleave the weak connection points within the 3DOM LSMO perovskite framework (Supplementary Fig. 3). The sonication cycle period totalled 12 h for a typical run with the yield (by weight) of 3D-hm particles being approximately 70% of the original 3DOM material used. As a final step, the collected supernatants were dried at 100 °C for 12 h to obtain the 3D-hm LSMO perovskites.

3D mesostructured LSMO comprising crystalline nanoparticles

A dissolution method was also assessed as a means of breaking the weak points within the 3DOM LSMO framework. The procedure gave undesirable agglomerated three-dimensionally mesostructured LSMO comprising crystalline nanoparticles (3D-aNPs LSMO) as shown in Supplementary Fig. 4. A typical dissolution process was performed in a Teflon

tube which contained 0.2 g of 3DOM LSMO sample. Basic L-lysine solutions with initial pH values in the range 9-10 and 10-11 were prepared with deionized water and added to the Teflon tube. Sonication was employed for the 3DOM LSMO sample for 10 min, allowing the large NPs to separate from each other before dissolution. The Teflon tube was then sealed and stored in a cool shady location for 4 days. The particles were then recovered by drying at 100 °C for 12h.

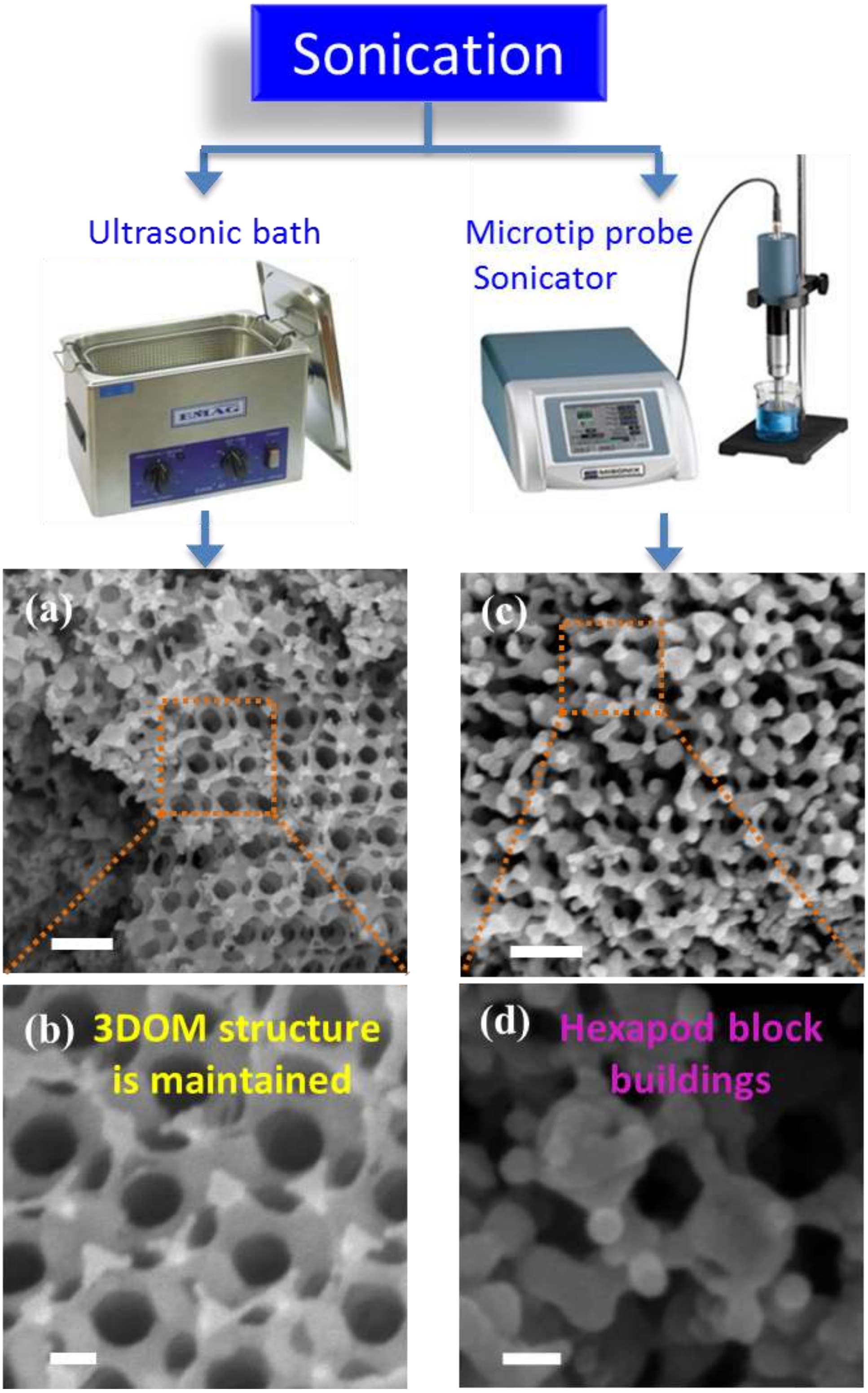

Supplementary Figure 3 FE-HRSEM images of LSMO samples synthesized by the sonication method (a, b) 3D-hm LSMO-1 prepared using an ultrasonic bath and (c, d) 3D-hm LSMO-2 prepared using a microtip probe sonicator. Scale bars in a and c are 200 nm. Scale bars in b and d are 50 nm.

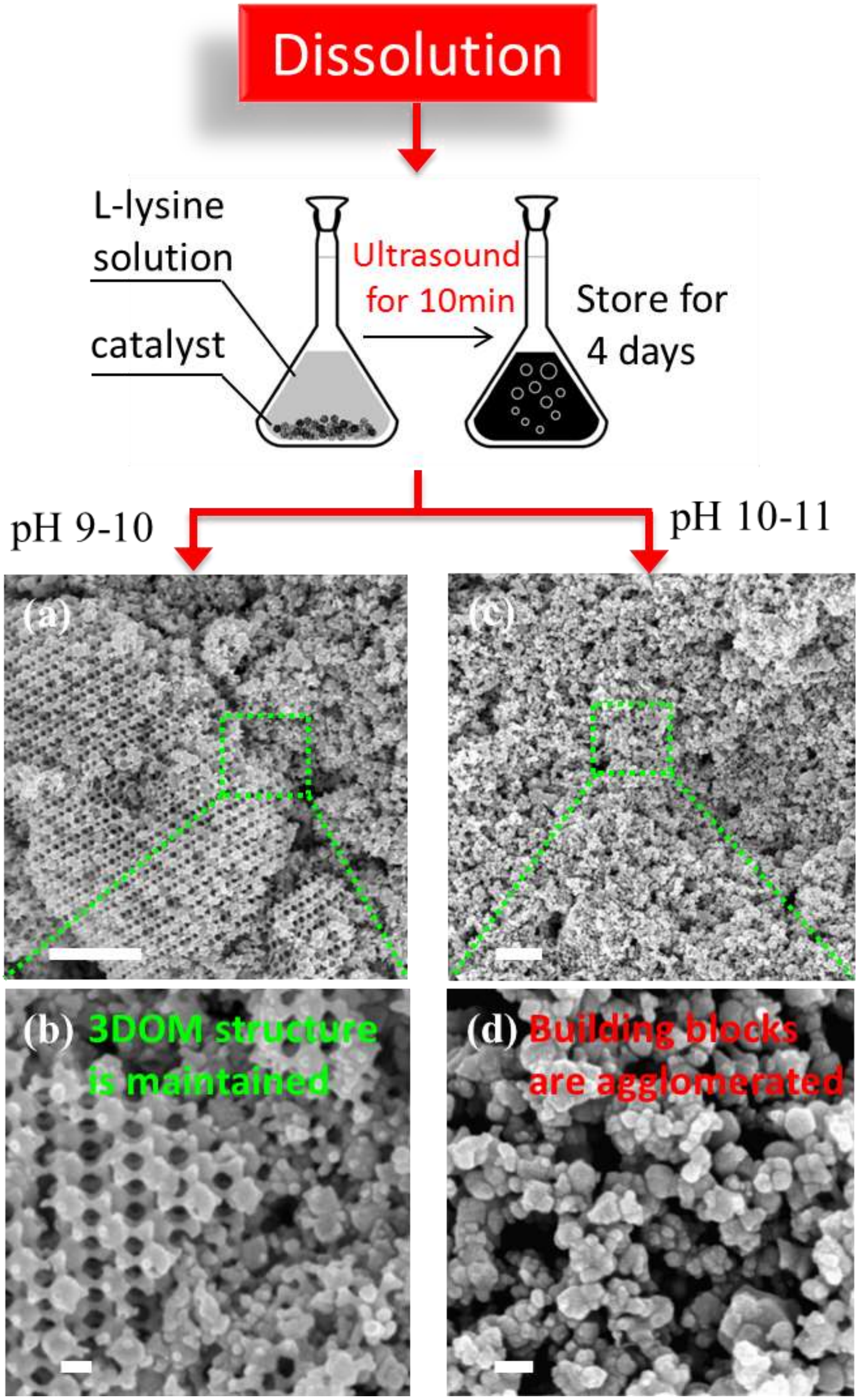

Supplementary Figure 4 FE-HRSEM images of LSMO samples synthesized by the dissolution method (a, b) 3D-aNPs LSMO-1 at pH 9-10 and (c, d) 3D-aNPs LSMO-2 at pH 11-12. Scale bars in a and c are 1 μm. Scale bars in b and d are 100 nm.

X-ray-diffraction (XRD) spectroscopy

X-ray diffraction (XRD) analyses were performed on a PANalytical Empyrean II diffractometer with Cu Kα radiation ($\lambda = 0.15406$ nm) at 45 kV and 40 mA to identify the crystal phases and to determine the lattice parameters. Scattering intensity was recorded in the range of $8^o < 2\theta < 90^o$ for all samples with a 2θ step of 0.03^o and a count time of 2 s per step. The diffraction patterns were indexed to JCPDS (Joint Committee on Powder Diffraction Standards) files.

Field-emission high-resolution scanning electron microscopy (FE-HRSEM)

Morphologies of the as-prepared samples were observed by scanning electron microscopy (SEM) and field-emission high resolution scanning electron microscopy (FE-HRSEM) using a FEI Nova NanoSEM 450 FE-SEM microscope at an accelerating voltage of 5 kV, with a work distance (WD) between 5 and 8 mm and magnifications in the range of 100−800000. HRSEM images were obtained using the back-scattered electron detector (BSED). The secondary electron detector (SED) modes show morphologies of the surface structures. To determine chemical compositions of the crystal phases, energy-dispersive spectroscopy (EDS) was used to obtain the EDS spectra by means of an EDS DX-4 analysis system. Prior to analysis the samples were coated with a thin Cr film to provide better conductivity of the sample, for example for the PMMA microspheres.

Field-emission high resolution transmission electron microscopy (FE-HRTEM)

Field-emission high resolution transmission electron microscopic (FE-HRTEM) images as well as the selected-area electron diffraction (SAED) patterns of the typical samples were obtained on a Philips CM200 apparatus. For identifying chemical compositions of the crystal phases, high-angle annular dark-field -scanning transmission electron microscopy energy-dispersive X-ray spectroscopy (HAADF-STEM-EDS) was employed using a JEOL JEM-

ARM200F STEM to obtain the EDS spectra.

Specific surface area and pore size distribution

The specific surface areas and pore size distributions were obtained using the N_2 physisorption method in conjunction with the Brunauer-Emmett-Teller (BET) and Barrett-Joyner-Halenda (BJH) models, respectively. The N_2 adsorption-desorption isotherms, surface areas, and pore diameters of the samples were determined via N_2 adsorption at –196 ^oC on a Micromeritics Tristar 3030 adsorption analyser. Before measurement, the samples were degassed at 150 ^oC for 3 h.

Oxygen temperature-programmed desorption (O_2 TPD)

For the O_2 TPD analysis, 20 mg of freshly prepared sample was loaded into a quartz U-shaped reactor and placed in a Micromeritics Autochem II apparatus equipped with a thermal conductivity detector (TCD) detector. The samples were initially treated with helium at a flow rate of 30 mL/min as the temperature was increased at a rate of 10 °C/min to 150 °C where it was held at this temperature for 30 min (to remove moisture) and then cooled to 50 °C. Then, pure O_2 at a flow rate of 30 mL/min was introduced to the sample, following by 30 mL/min helium flushing. After 30 min equilibrium, the temperature ramped at ramp rate of 10 °C/min from room temperature to 900 °C. The variation in O_2 concentration from the cell effluent was monitored on-line by the TCD detector.

X-ray photoelectron spectroscopy (XPS)

X-ray photoelectron spectroscopy (XPS) was used to determine the La 3d, Mn $2p_{3/2}$, O 1s, and C 1s binding energies (BEs) of surface species. The analyses were performed on a Thermo Scientific, UK (model ESCALAB250Xi) using Mg Kα ($h\nu$ = 1486.68 eV) as the excitation source with 150W power (13 kV x 12 mA). Before XPS measurement, the sample was treated in an O_2 flow of 20 mL/min at 200 ^oC for 1 h. After cooling to RT, the pretreated

sample was transferred to a holder in a Glove Bag (Instruments for Research and Industry, USA) that was filled with helium, and then the holder was transferred into the spectrometer chamber under helium. The sample was outgassed (0.5 h) in the preparation chamber before being analysed in the analysis chamber. The BE values of La 3d, Mn $2p_{3/2}$, O 1s, and C 1s were calibrated against the C 1s signal (BE = 284.6 eV for adventitious hydrocarbon) of contaminant carbon with a spectrometer calibration of Au 4f7 = 83.96 eV, Ag 3d5 = 368.21 eV, Cu 2p3 = 932.62 eV.

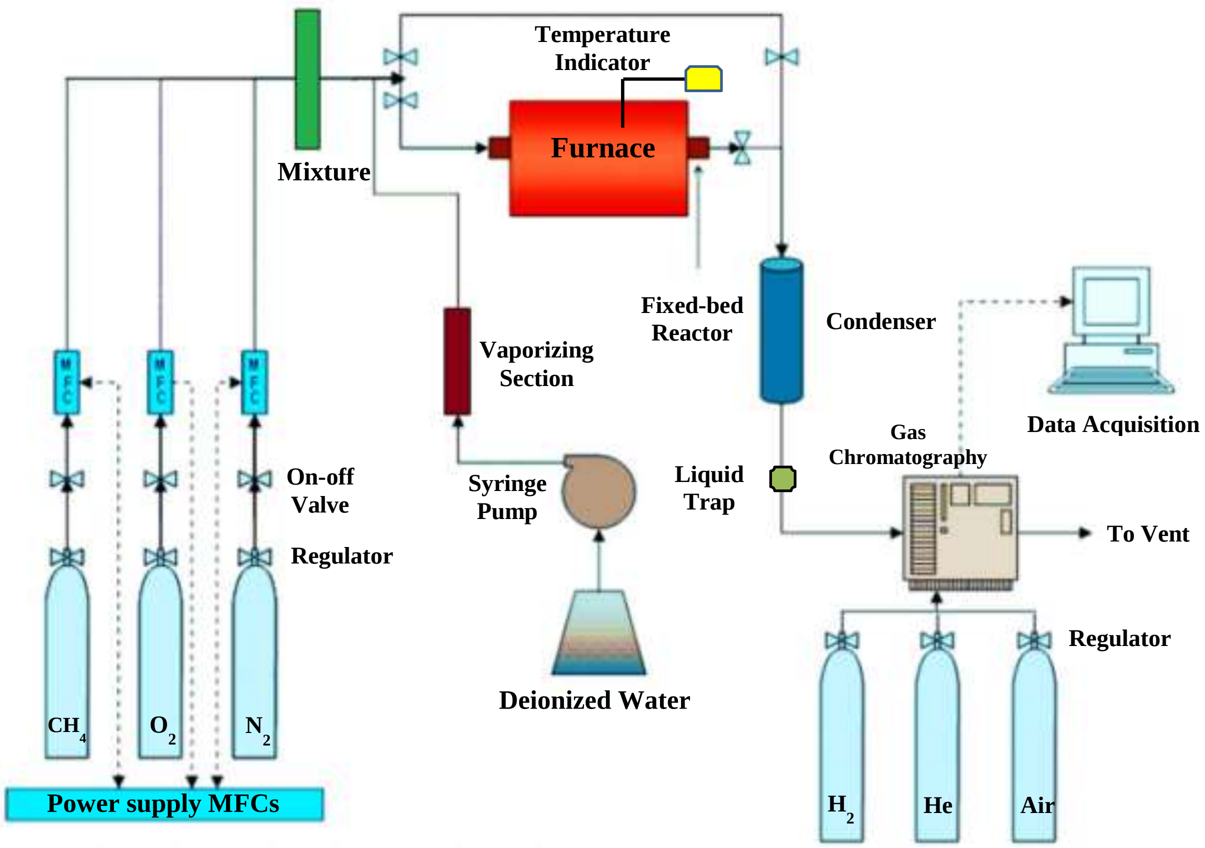

Supplementary Figure 5 Illustration depicting the catalytic rig set-up.

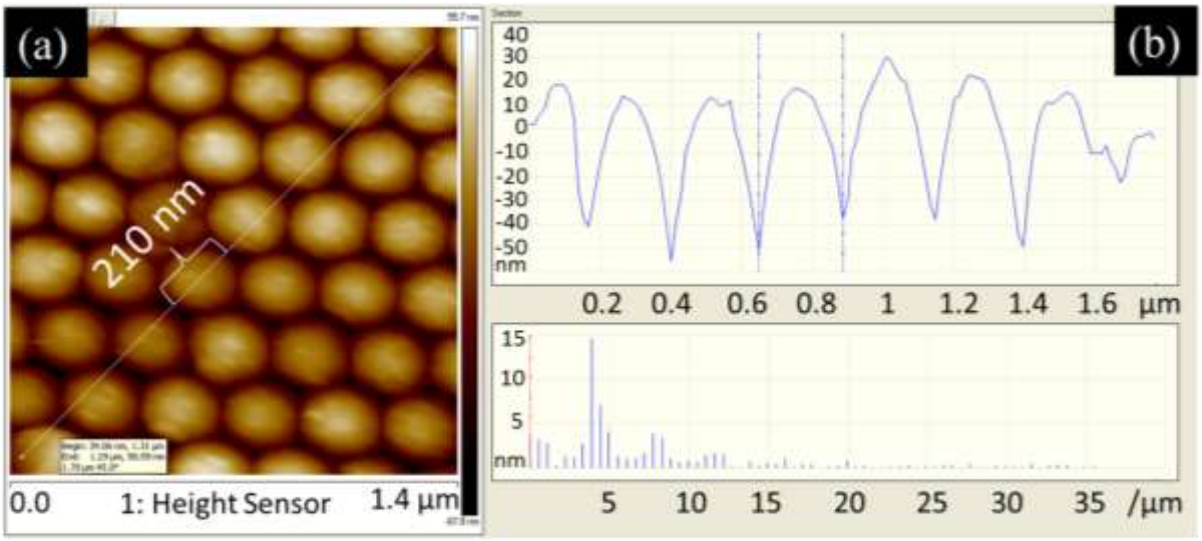

Supplementary Figure 6 (a) 3D-eAFM image and (b) the corresponding cross-sectional profile of the periodic hexagonal close-packed single-layered monodisperse PMMA spheres.

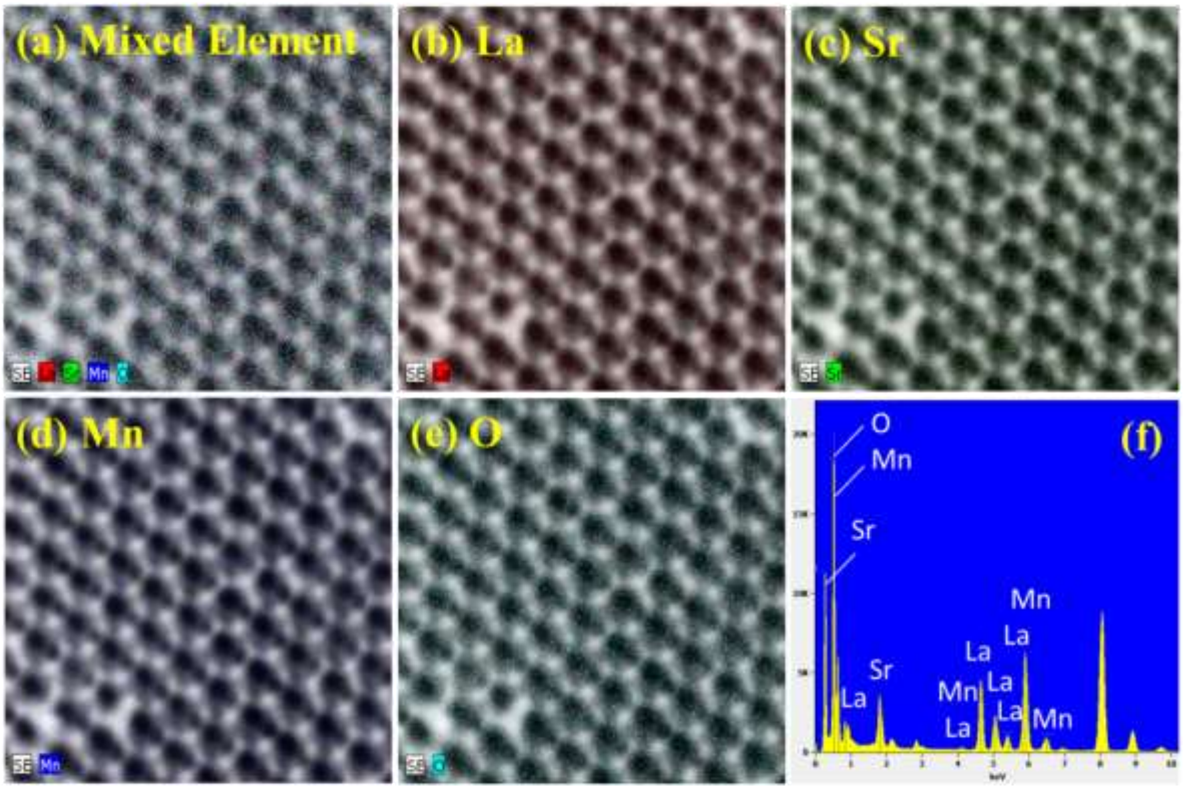

Supplementary Figure 7 EDS elemental mapping images of (a) all elements over-layed, (b) La, (c) Sr, (d), Mn, and (e) O and (f) the EDS spectrum of the 3DOM LSMO sample calcined at 750 °C.

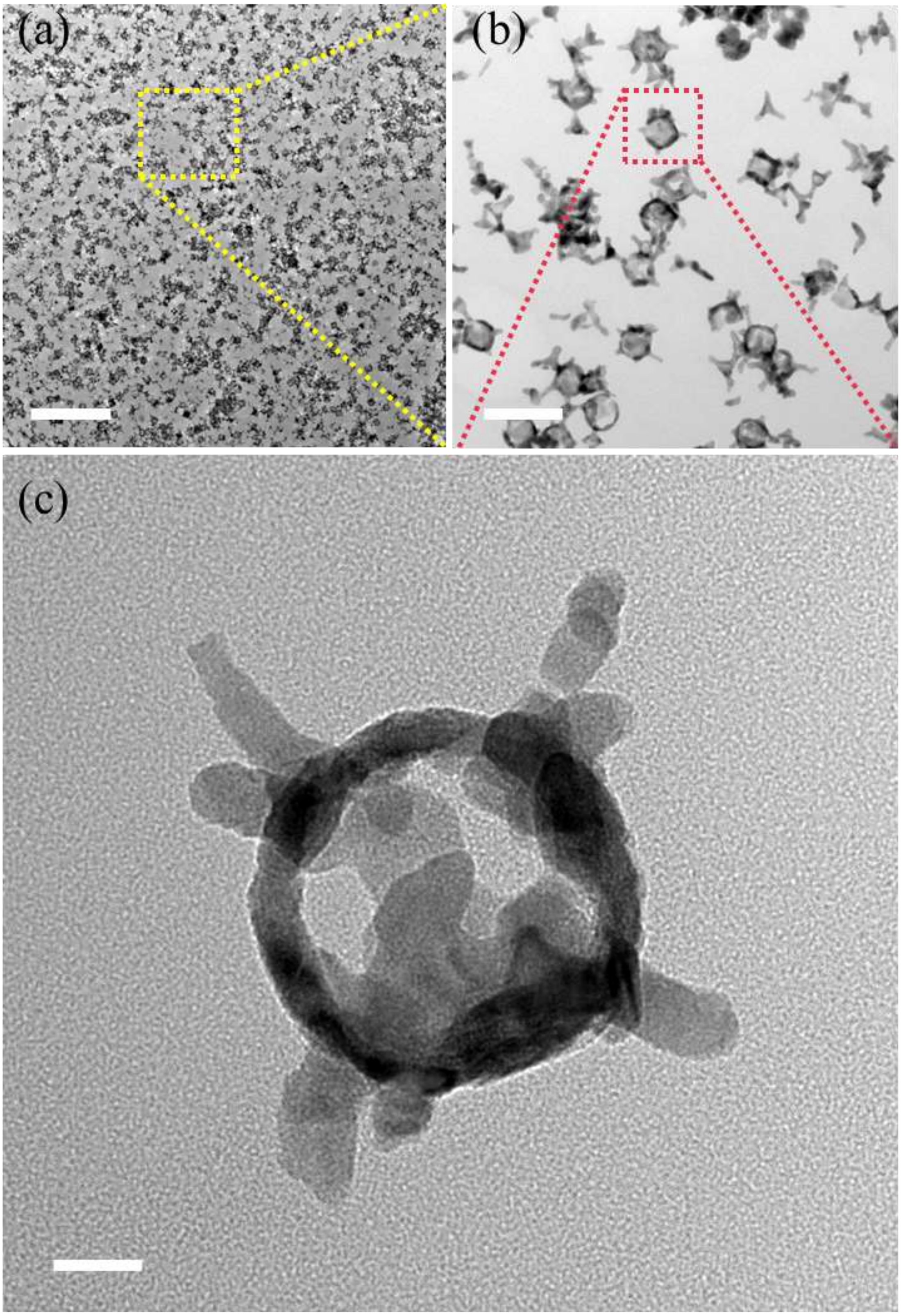

Supplementary Figure 8 FE-HRTEM images of 3D-hexopod LSMO at different magnifications. Scale bars in a, b and c are 1 μm, 200 nm and 20 nm, respectively.

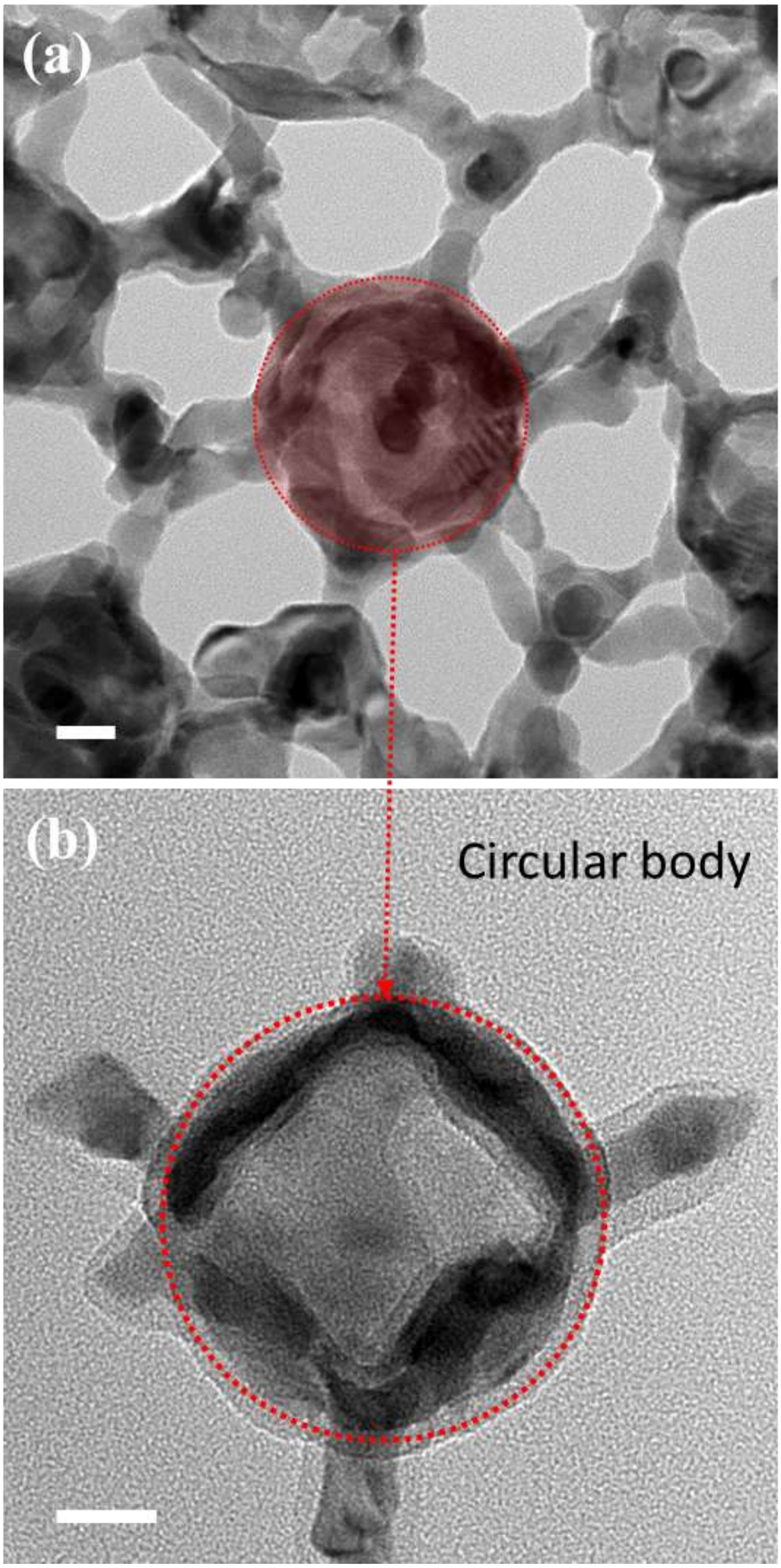

Supplementary Figure 9 FE-HRTEM images of (a) 3DOM LSMO and (b) 3D-hm LSMO possessing a circular-shaped body. Scale bars in a and b are 20 nm.

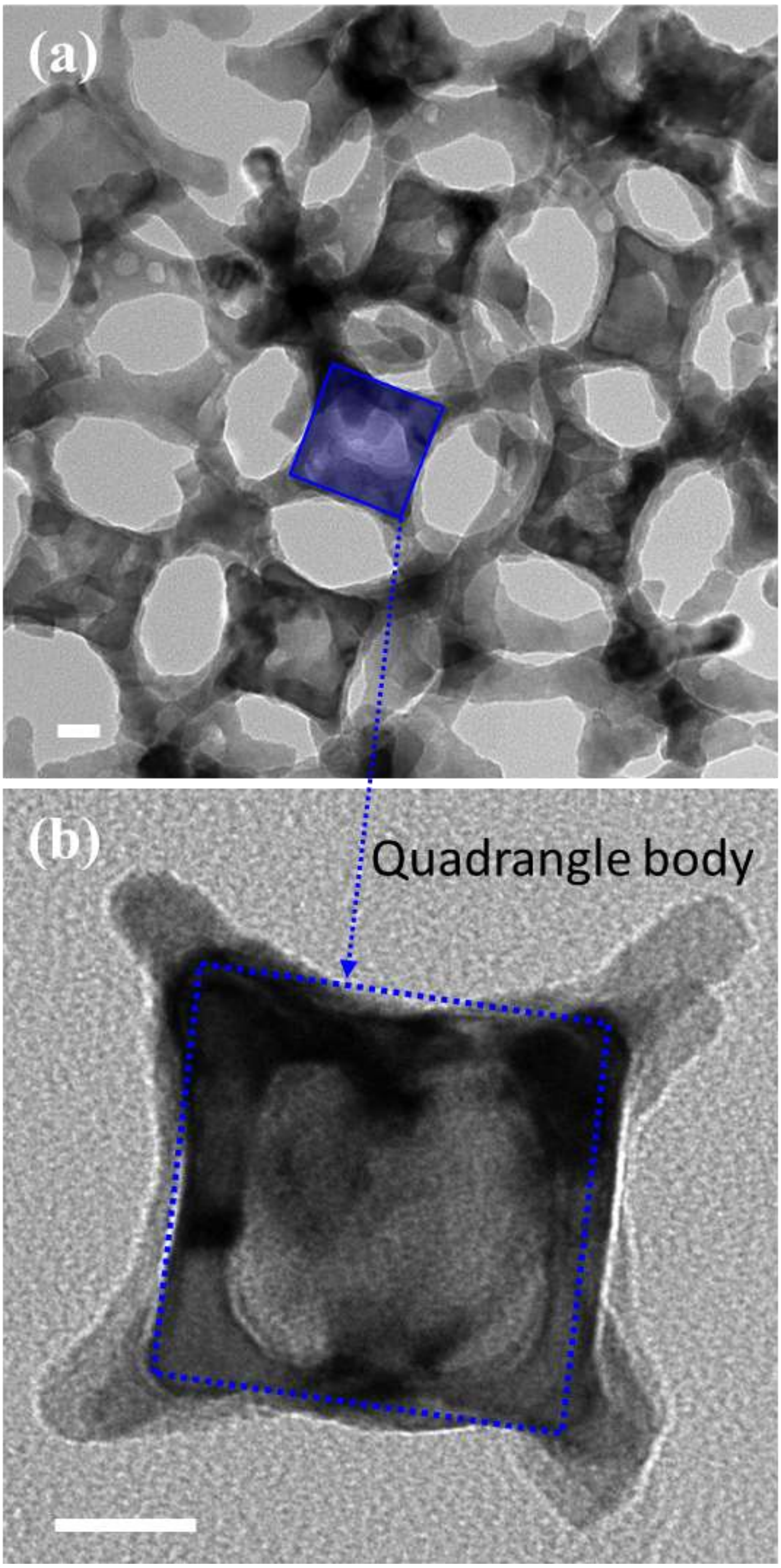

Supplementary Figure 10 FE-HRTEM images of (a) 3DOM LSMO and (b) 3D-hm LSMO possessing a quadrangle-shaped body. Scale bars in a and b are 20 nm.

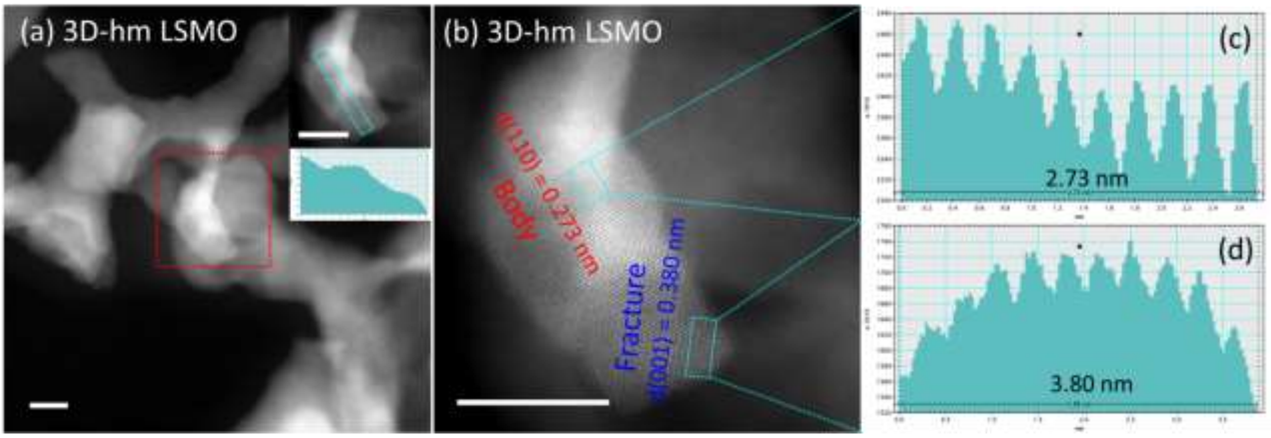

Supplementary Figure 11 HAADF-STEM images of 3D-hm LSMO. Scale bars in a and b are 10 nm.

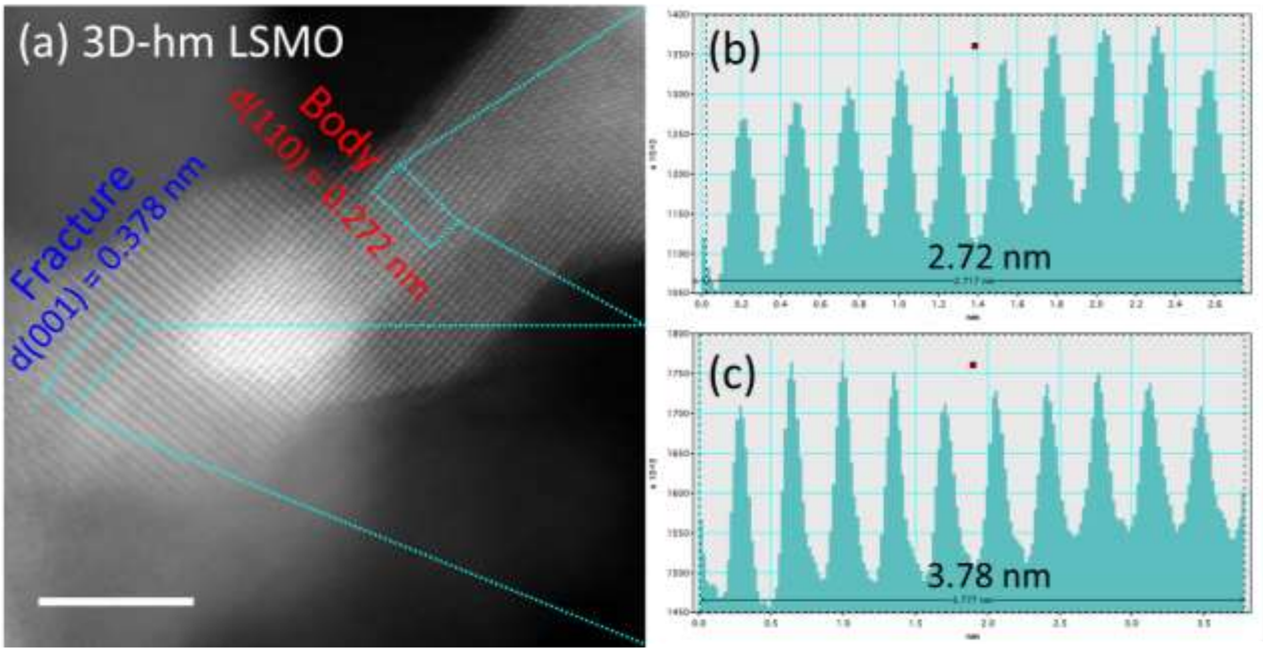

Supplementary Figure 12 HAADF-STEM images of 3D-hm LSMO. Scale bar in a is 5 nm.

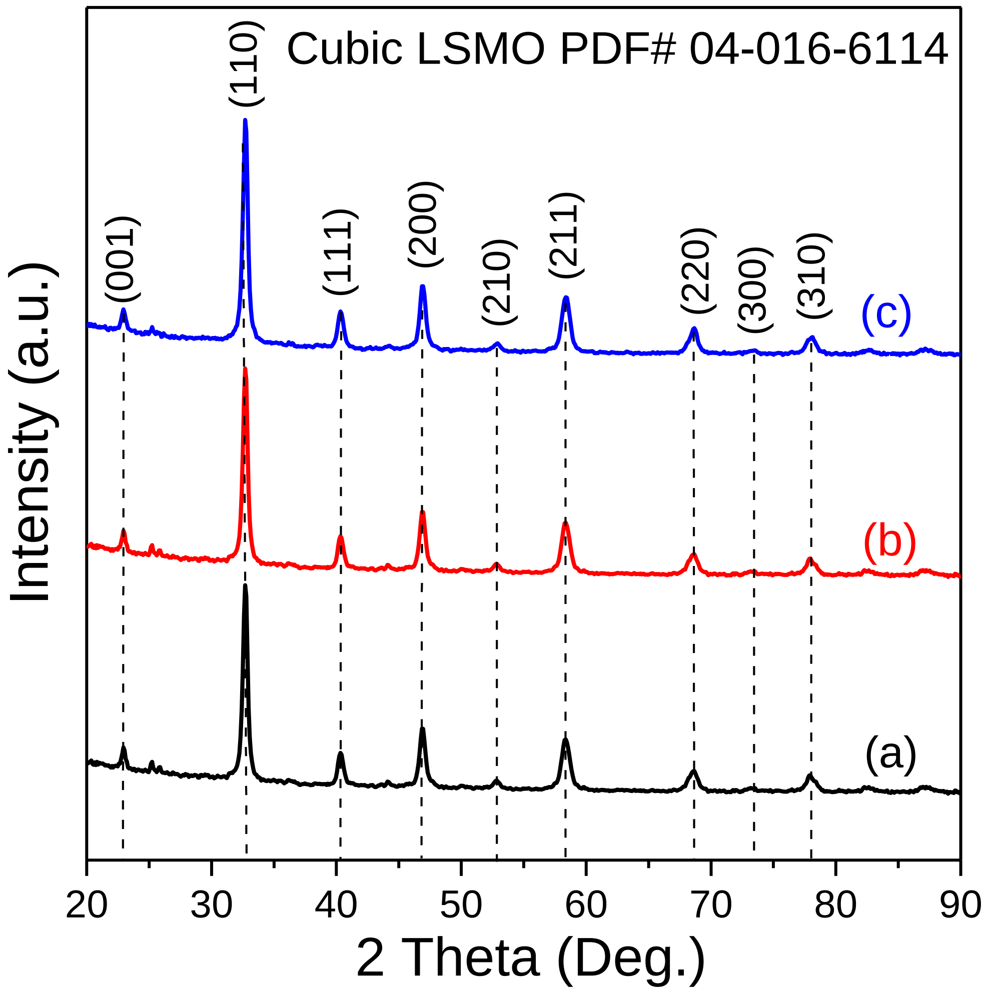

Supplementary Figure 13 XRD profiles of (a) 1DDN LSMO (b) 3DOM LSMO and (c) 3D-hm LSMO.

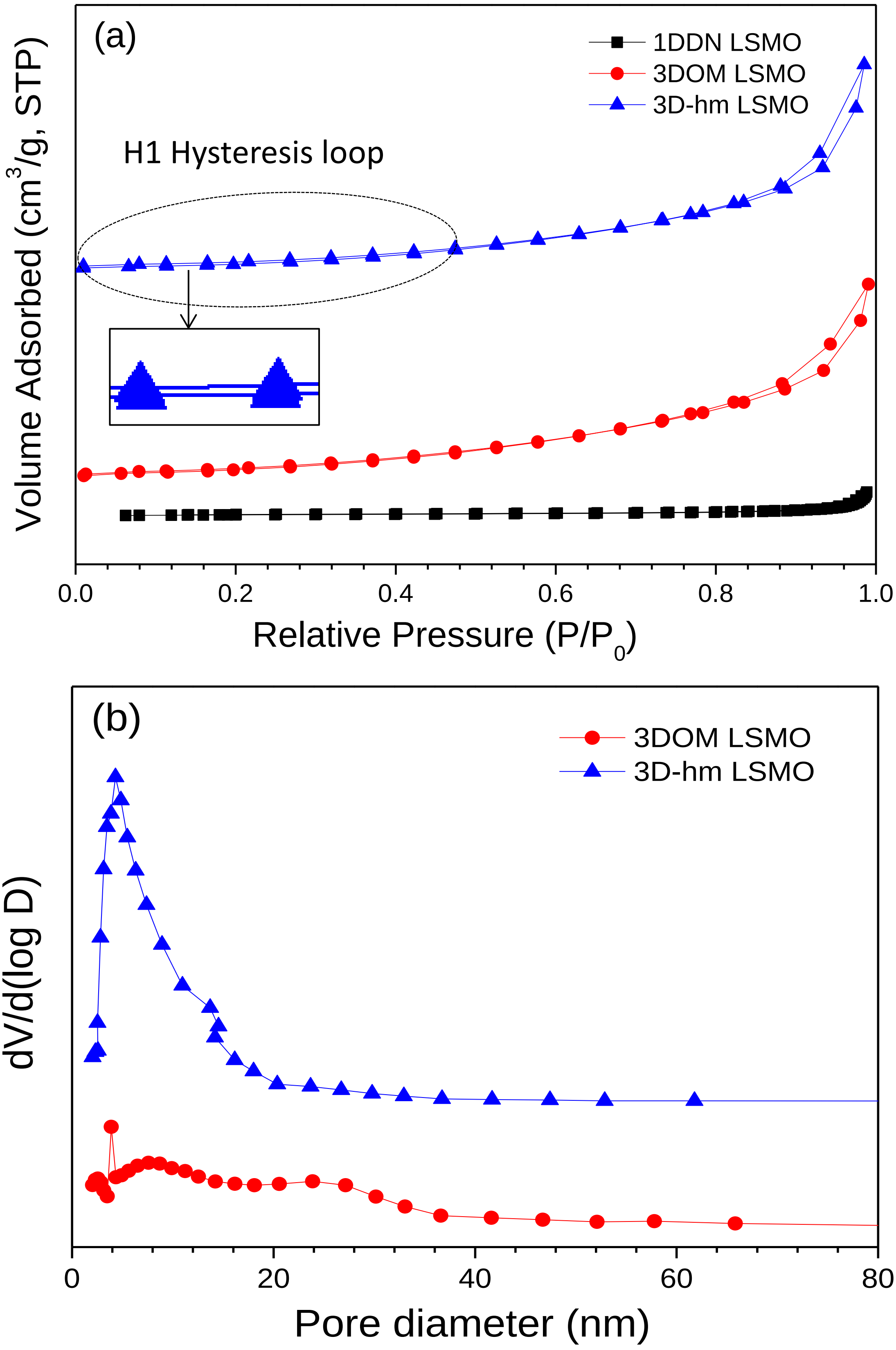

Supplementary Figure 14 (a) Nitrogen adsorption–desorption isotherms and (b) pore-size distributions of the LSMO samples.

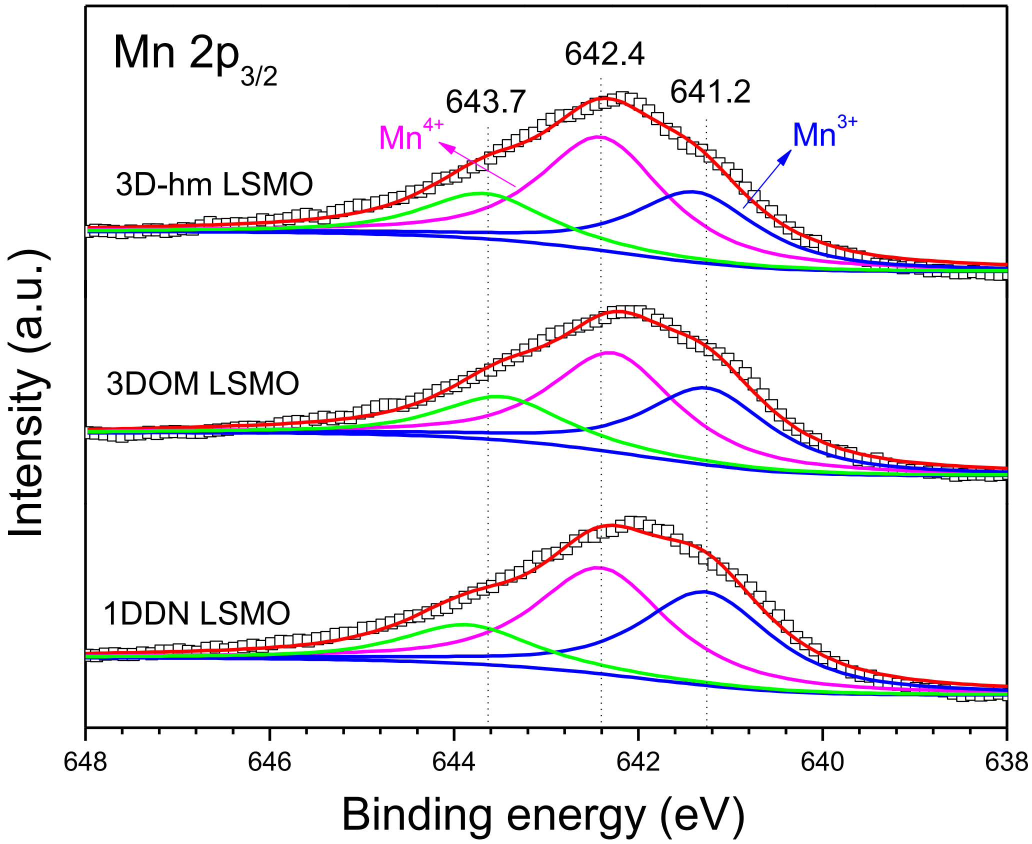

Supplementary Figure 15 Mn $2p_{3/2}$ XPS spectra of the LSMO samples.

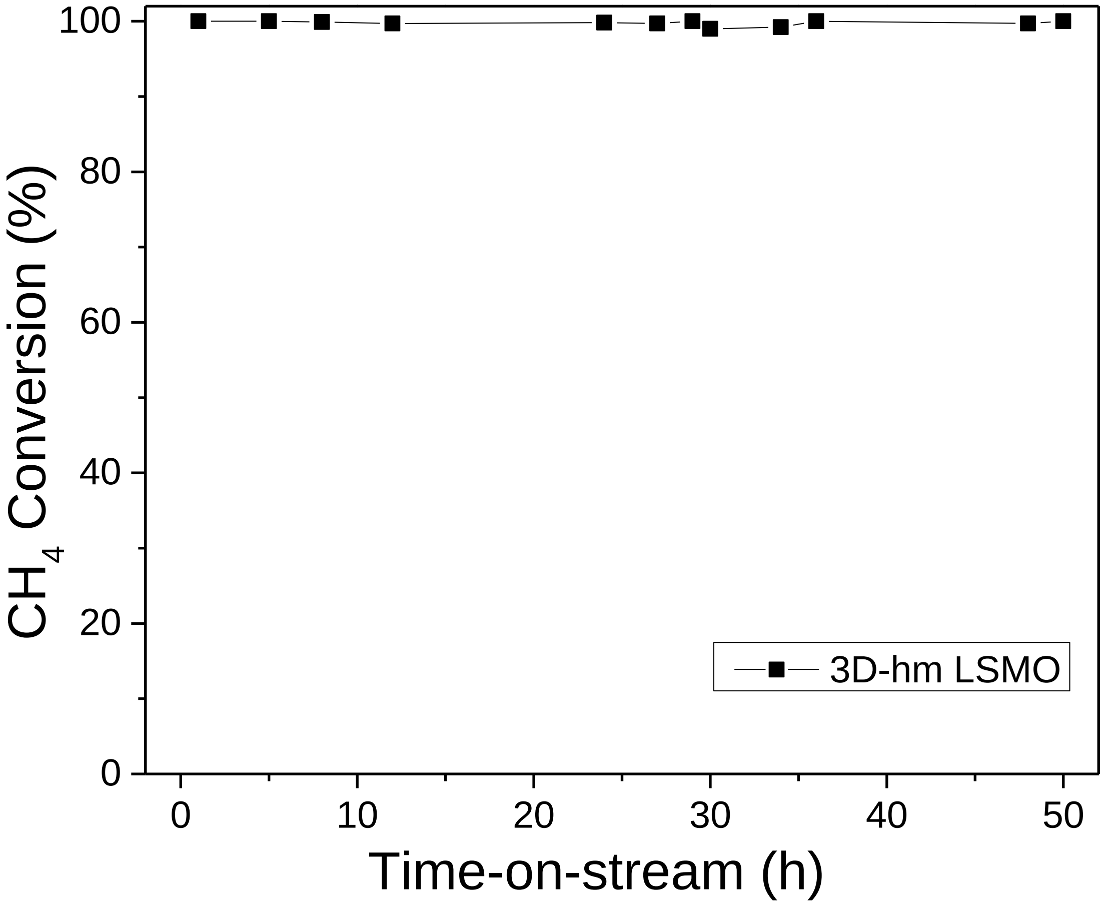

Supplementary Figure 16 Stability test for 3D-hm LSMO: CH_4 conversion at 650 °C during 50 h on-stream reaction at a GHSV = 50 000 mL/(g h) under the conditions of 5 vol% CH_4 + 30 vol% O_2 + 65 vol% N_2 with a total flow rate = 42.8 mL/min.

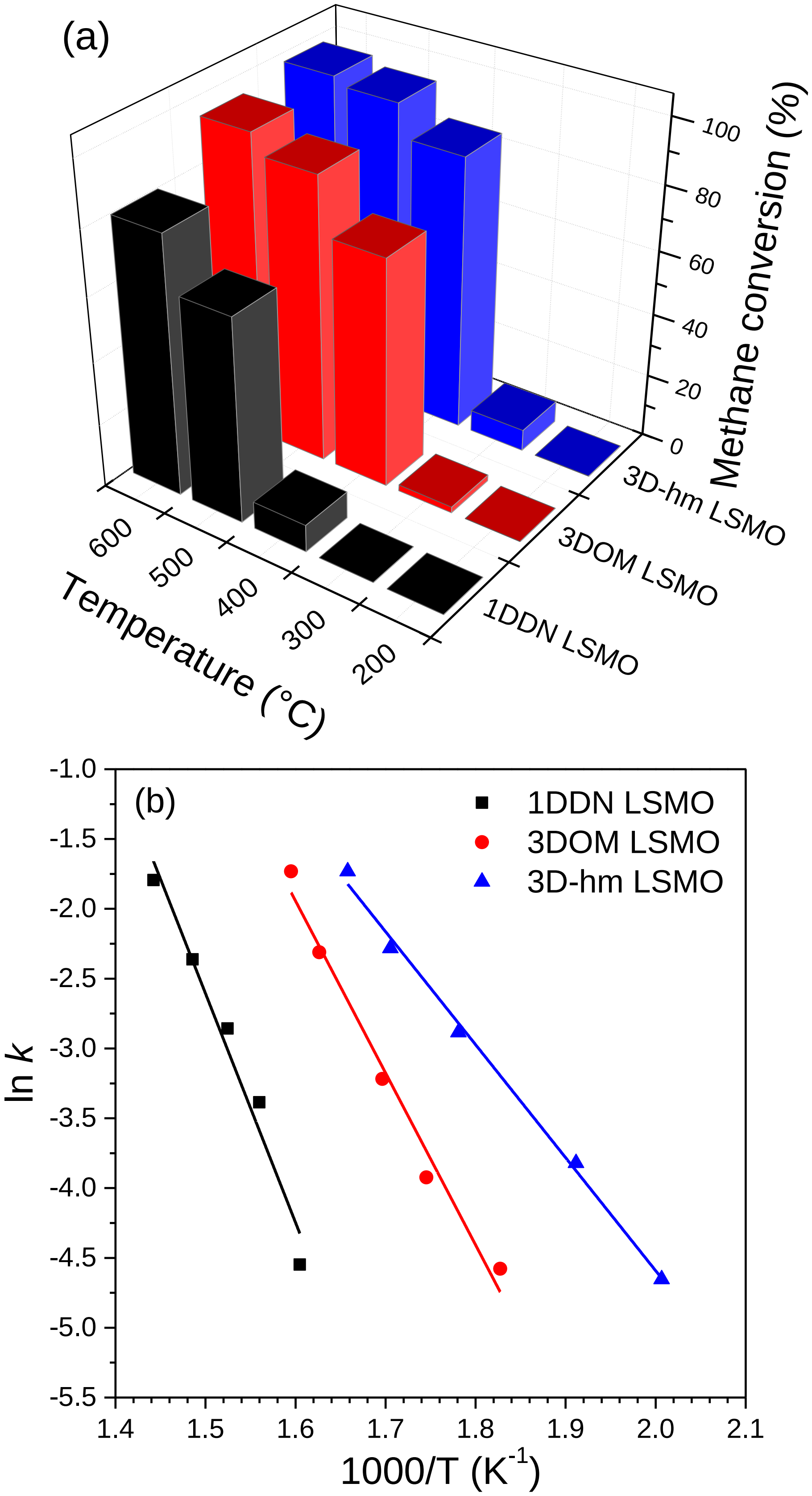

Supplementary Figure 17 (a) Effect of LSMO morphology on catalytic activity and (b) Arrhenius plots of methane conversion by the as-prepared catalysts.

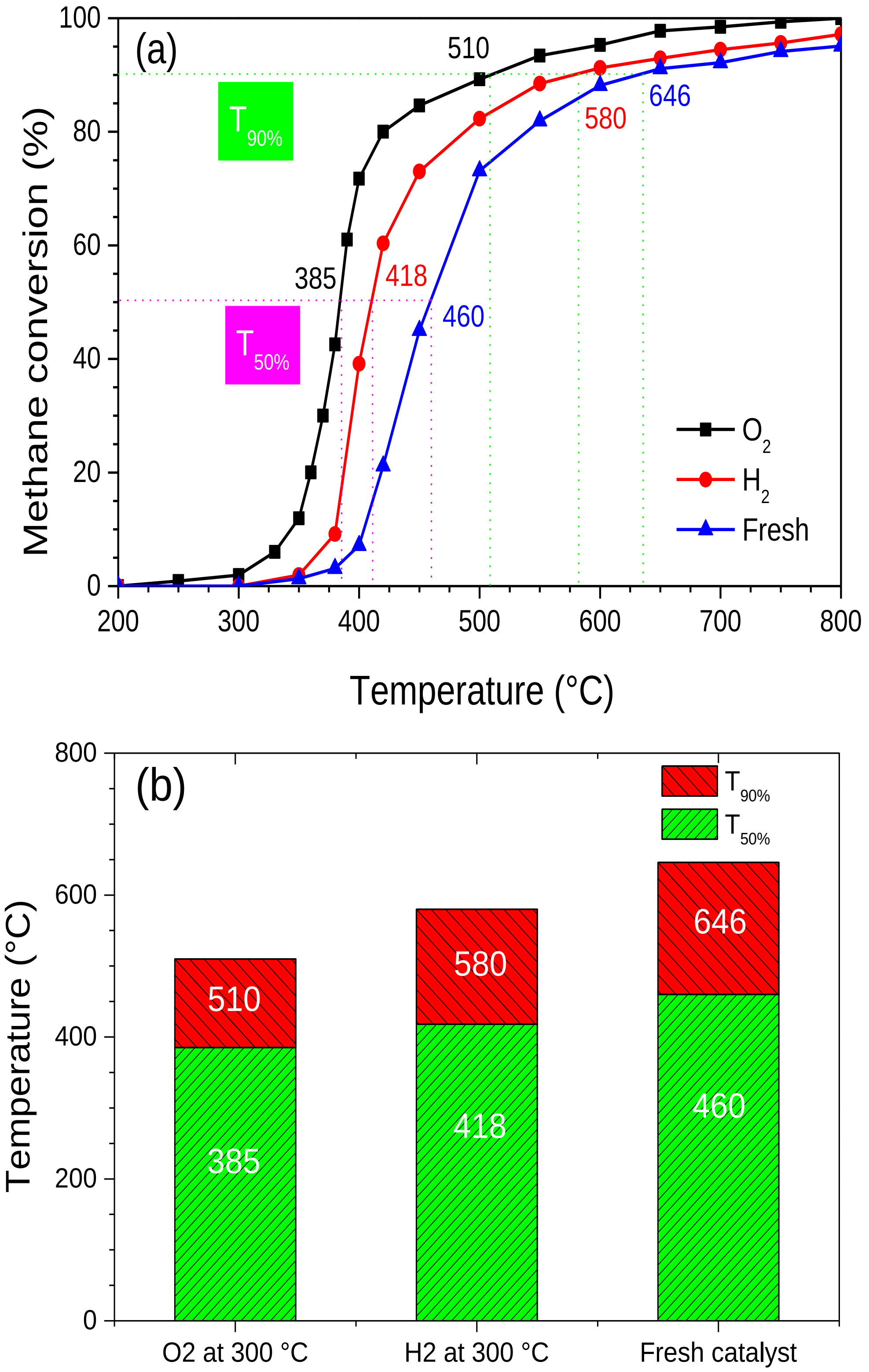

Supplementary Figure 18 Effect of catalyst activation strategy (reduction and oxidation) on CH_4 conversion by the 3DOM LSMO sample.

The oxidised $Mn^{\delta+}$ species are observed to be more active than the metallic (i.e. reduced) Mn^0 species for methane oxidation (Supplementary Fig. 18).

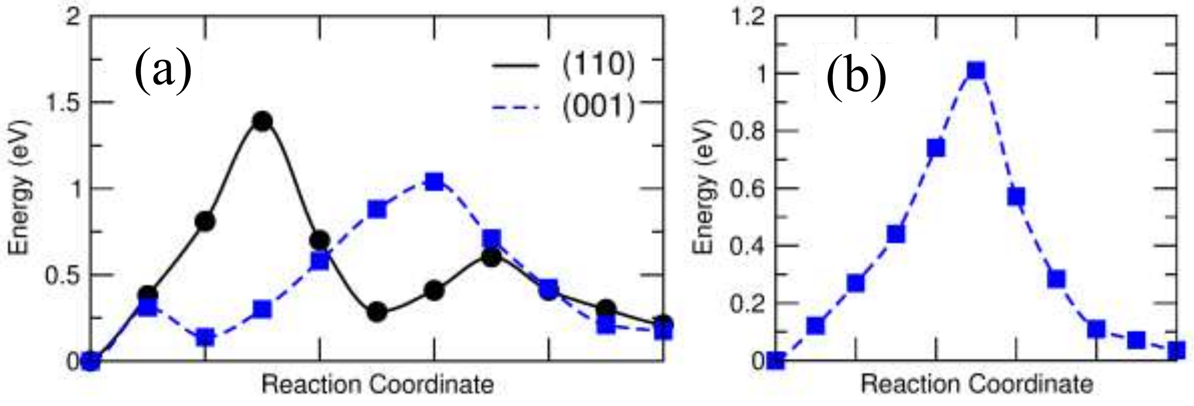

Supplementary Figure 19. Lowest energy dissociation barriers for CH_4*→CH_3*+H* on the (001) and (110) LSMO surfaces.

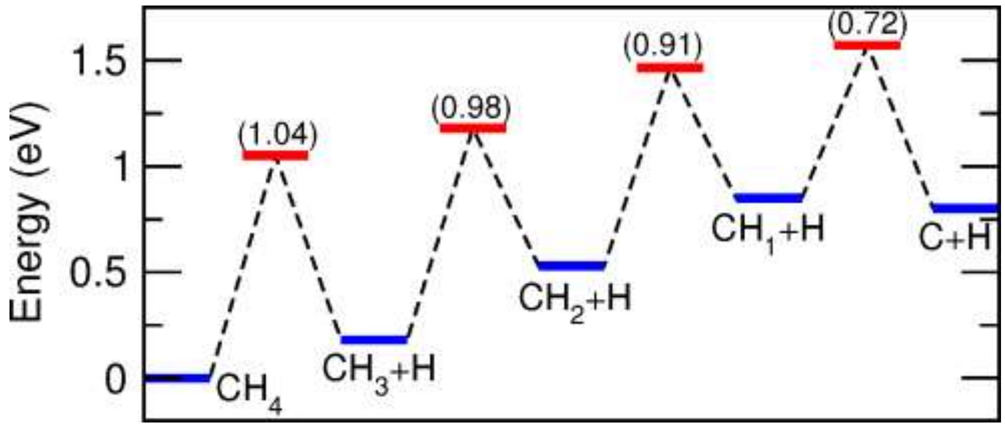

Supplementary Figure 20 Schematic profile of the energetics of the successive CH_4 dehydrogenation steps on the (001) LSMO surface. The activation barrier is shown in parenthesis.

Supplementary Discussions

Theoretical methodology

The (001) and (110) surfaces were cleaved from an optimized cubic pm$\overline{3}$m $LaMnO_3$. Various terminations can arise for the (001) and (110) surfaces. For the (001) orientated surfaces the stacking of the layers follow $AO/BO_2/AO/BO_2$, while for the (110) surfaces the stacking is $ABO/O_2/ABO/O_2$. While both surfaces are non-dipolar, those for (110) have a natural centre of symmetry that constrains the dipole to remain zero during relaxation. However, for (001) relaxation can lead to a net dipole due to asymmetry of the flat surfaces. Therefore it is necessary to reconstruct the (001) surface to preserve the symmetry. This can be achieved by a 50% occupancy of the AO layer on both sides of the slab. An alternative (001) surface can be created with a similar partial occupancy for BO_2 layers on both sides. For (001) surfaces, the BO_2 termination was focussed on as this is expected to be the most catalytically active surface. For (110) both terminations (ABO or BO_2 surface layers) were considered. Adsorption energies were calculated using symmetric (9 layer) and asymmetric (8 layer) slabs. To mimic the random distribution of Sr in LSMO, special quasi-random structures (SQS) were used. These were generated using the Monte Carlo SQS tool within the Alloy Theoretical Automated Toolkit (ATAT) [2]. The binding energies of methane at various sites were calculated using Supplementary Equation 1:

$$E_b(CH_4) = E_{tot}(LSMO/CH_4) - E_{tot}(LSMO) - E_{tot}(CH_4) \quad (1)$$

where $E_{tot}(LSMO/CH_4)$ is the total energy of the combined system, while $E_{tot}(LSMO)$ and $E_{tot}(CH_4)$ are the total energies of the bare LSMO slab and an isolated CH_4 molecule, respectively. The dissociation barriers were calculated using the climbing image (CI) nudged elastic band method [3] with nine images interpolating between the relaxed initial and final

configurations. Migration barriers were lower on the (001) surface than the (110) surface. Below are the lowest energy paths on each surface.

Supplementary Fig. 19a shows the presence of metastable sites along the migration pathway. To further resolve the activation barrier on the (001) surface we considered the metastable site as the starting point for the CI-NEB calculation (Supplementary Fig. 19b). Using nine images the refined pathway is about 1.01 eV which is very close to the value obtained originally.

The successive dehydrogenation steps on the (001) surface are shown in Supplementary Fig. 20. The first dehydrogenation step has a higher energy barrier compared to the other steps and thus, constitutes the rate determining step. This observation is in agreement with previous theoretical studies. [4-7] However, the activation barriers are close in energy to each other which could imply the presence of multiple rate determining steps.

Weisz-Prater analysis

The mass and heat transfer limitations were studied by selecting the most active 3D-hm LSMO catalyst at the CH_4 conversion of 50 % and temperature 360 ^{o}C with a GHSV of 50 000 mL/(g h). Supplementary Table 1 lists the parameters used in the calculations. The absence of internal mass diffusion transport resistance was checked by the Weisz-Prater Criterion ($N_{W\text{-}P}$)[8].

If $N_{W-P} = \dfrac{r_a \cdot \rho_c \cdot R_p^2}{C_s \cdot D_{eff}} \leq 1$, internal mass transfer effects can be neglected.

$$N_{W-P} = \frac{r_a \cdot \rho_c \cdot R_p^2}{C_s \cdot D_{eff}} = \frac{1.63\times10^{-5}\times5.0\times10^{6}\times(8\times10^{-8})^2}{20.5\times4.89\times10^{-6}} = 5.2\times10^{-9} << 1 \qquad (2)$$

If $\frac{r_a \cdot \rho_b \cdot R_p \cdot n}{k_c \cdot C_{sb}} \leq 0.15$, external mass transfer effects can be neglected.

$$\frac{r_a \cdot \rho_c \cdot R_p \cdot n}{k_c \cdot C_{sb}} = \frac{1.63\times10^{-5} \times 2.5\times10^{6} \times 8\times10^{-8} \times 1}{1\times 40.9} = 7.9\times10^{-8} << 0.15 \qquad (3)$$

Based on the above analyses, no significant mass transfer limitation are present under the utilised reaction conditions.

Reaction rate calculation

The method for estimating metal oxide specific reaction rate was similar to that reported by Liu *et al.*[8]

$$r_1 = \frac{r_2 \times N}{100\%} \qquad (4)$$

$$r_a = r_1/m \qquad (5)$$

$$r_a' = \frac{r_a}{s} \qquad (6)$$

Here we use 3D-hm LSMO as an example. 50 mg of 3D-hm LSMO has a total surface area of 48.9 m^2/g. An influent 5 vol% CH_4 gas stream at a total flow of 42.8 mL/min gives a GHSV of ca. 50 000 mL/(g h). The conversion rate of CH_4 over 3D-hm LSMO at 375 °C is 74.0%. For 3D-hm LSMO:

$$r_a = \frac{r_2 \times N}{m \times 100\%} = \frac{74.0\% \times 1.60 \times 10^{-6}}{5 \times 10^{-2} \times 100\%} = 2.36 \times 10^{-5}\ \text{mol}\ g_{cat}^{-1}\ s^{-1} \qquad (7)$$

$$r_a' = \frac{r_a}{s} = \frac{27.6}{48.9} = 4.84 \times 10^{-7}\ \text{mol}\ m^2\ s^{-1} \qquad (8)$$

Crystallite size

The average crystallite sizes (D) of the LSMO samples were estimated by applying the Debye–Scherrer equation to the most prominent line of the XRD data: $D(hkl) = 0.89\lambda/(\beta \cos \theta)$, where β is the half-height width of the diffraction peak of the sample, θ is the diffraction angle, and λ is the X-ray wavelength corresponding to the Cu $K\alpha$ radiation. Lattice parameters of the catalysts were calculated by a standard cubic indexing method using the intensity of the most prominent (110) peak. As seen in Supplementary Table 2, the crystallite sizes of the sonicated samples (3D-hm LSMO) remained unchanged compared with the original 3DOM LSMO, while the samples subjected to dissolution grew slightly from 35.7 nm to 36.9 nm (which is larger than 1DDN LSMO sample (36.2 nm)).

Supplementary Table 1. The parameters used in reaction rate, Weisz-Prater analysis and Mears analysis of LSMO catalysts.

Items	Unit	Description
r_1	mol/s	Reacting moles of CH_4 per second
r_2	%	Conversion of CH_4
N	mol/s	Initial CH_4 flow rate
r_a	mol/g_{cat} s	Specific reaction rate of CH_4 oxidation
r_a'	mol/m^2 s	Specific reaction rate of CH_4 oxidation
s	m^2/g	Catalyst surface area
m	g	Catalyst mass
R_p	m	Radius of catalyst particle
C_s	mol/m^3	Gas concentration of CH_4 at the catalyst surface
D_{eff}	m^2/s	Effective gas-phase diffusivity
ρ_c	g/m^3	Density of solid catalyst
ρ_b	g/m^3	Bulk density of catalyst bed
n	/	Reaction order
k_c	m/s	Estimated mass transfer coefficient
C_{sb}	mol/m^3	Bulk gas concentration of CH_4

Supplementary Table 2. Preparation parameters, specific surface area, crystal type and crystallite sizes (*D*) of 1DDN LSMO, 3DOM LSMO and disassembled 3DOM LSMO samples.

Catalyst code	Preparation Method	Preparation Conditions	BET Surface area [a] (m^2/g)	XRD	
				Crystal type	*D* [b] (nm)
1 DDN LSMO	Citrate	Calcined at 750 ºC in Muffle furnace	4.30	Cubic	36.2
3DOM LSMO	Colloidal crystal templating	Calcined using two steps in a tube furnace	33.5	Cubic	35.7
3D-hm LSMO-1	Sonication	Ultrasonic bath	37.2	Cubic	35.1
3D-hm LSMO-2	Sonication	Microtip probe	49.0	Cubic	35.5

		sonicator			
3D-aNPs LSMO-1	Dissolution	L-lysine solution at pH 9-10	39.4	Cubic	36.1
3D-aNPs LSMO-2	Dissolution	L-lysine solution at pH 10-11	26.2	Cubic	36.9

[a] data was obtained using N_2 physisorption analysis and BET model.
[b] data was obtained from the XRD results according to the Scherrer equation.

Supplementary Table 3. Pore volume, pore diameter, H_2 consumption and surface composition of 1DDN LSMO, 3DOM LSMO and 3D-hm LSMO samples.

Catalyst	Pore Volume [a.] (cm^3/g)	Pore diameter [b.] (nm)	XPS	
			O_{ads}[c]/O_{latt}[d] **molar ratio**	**La/Mn molar ratio**
1DDN LSMO	0.0167	-	0.30	0.50
3DOM LSMO	0.1051	140	0.35	0.44
3D-hm LSMO	0.1521	5-40	0.44	0.43

[a] The data was obtained by BET analysis.
[b] The data was determined by SEM results.
[c] Adsorbed oxygen species.
[d] Lattice oxygen species.

Supplementary Table 4. Temperatures at which 90% methane conversion was achieved ($T_{90\%}$) and activation energies (E_a) for methane oxidation catalysts used in this study and reported in the literature.

Catalyst	Structure	Reaction condition	$T_{90\%}$ (°C)	E_a (kJ/mol)	Ref.
$La_{0.6}Sr_{0.4}MnO_3$	3D-hexapod mesostructured	$5\%CH_4+30\%O_2+65\%N_2$, GHSV=50,000 mL/(g h)	438	77.9	Present work
$La_{0.6}Sr_{0.4}MnO_3$	3D ordered macroporous	$5\%CH_4+30\%O_2+65\%N_2$, GHSV=50,000 mL/(g h)	510	94.7	Present work
$La_{0.6}Sr_{0.4}MnO_3$	1D disordered nonporous	$5\%CH_4+30\%O_2+65\%N_2$, GHSV=50,000 mL/(g h)	690	125	Present work
$La_{1.0}Co_{0.96}Fe_{0.003}O_{3-\Delta}$	Nanoparticles	$0.25\%CH_4+98.75\%O_2+1\%Ne$, GHSV=22,500 mL/(g h)	610	102.5	9
$La_{0.91}Co_{0.92}Fe_{0.17}O_{3-\Delta}$	Nanoparticles	$0.25\%CH_4+98.75\%O_2+1\%Ne$, GHSV=22,500 mL/(g h)	470	106.7	9
$La_{0.99}Fe_{1.01}O_{3-\Delta}$	Nanoparticles	$0.25\%CH_4+98.75\%O_2+1\%Ne$,	505	93.7	9

		GHSV=22,500 mL/(g h)			
$La_{0.7}Sr_{0.1}Ce_{0.2}FeO_3$	Nanoparticles	$4.35\%CH_4+8.70\%O_2+86.95\%He$, GHSV=70,000 mL/(g h)	725	83.9	[10]
$La_{0.5}Sr_{0.2}Ce_{0.3}FeO_3$	Nanoparticles	$4.35\%CH_4+8.70\%O_2+86.95\%He$, GHSV=70,000 mL/(g h)	750	91.0	[10]
$La_{0.8}Ce_{0.2}FeO_3$	Nanoparticles	$4.35\%CH_4+8.70\%O_2+86.95\%He$, GHSV=70,000 mL/(g h)	-	84.2	[10]
$Ni_{0.5}Co_{2.5}O_4$	Nanoarrays	$1\%CH_4+20\%O_2+9\%N_2+70\%Ar$, GHSV=45,000 mL/(g h)	575	89	[11]
Co_3O_4	Nanoarrays	$1\%CH_4+20\%O_2+9\%N_2+70\%Ar$, GHSV=45,000 mL/(g h)	640	124	[11]
$Zn_{0.5}Co_{2.5}O_4$	Nanoarrays	$1\%CH_4+20\%O_2+9\%N_2+70\%Ar$, GHSV=45,000 mL/(g h)	705	220.4	[11]
$Mn_{0.6}Ce_{0.4}$	Nanorods	$0.5\%CH_4+3\%O_2+96.5\%He$, GHSV=36,000 mL/(g h)	630	-	[12]
$Mn_{0.3}Ce_{0.7}$	Nanorods	$0.5\%CH_4+3\%O_2+96.5\%He$, GHSV=36,000 mL/(g h)	710	-	[12]

Supplementary Table 5. Adsorption energies of CH_4 on various sites on the (001) and (110) surface.

		**Binding energy (eV)**	
**System**	**Configuration**	**PBE**	**D3**
(001) SrO termination	CH4@O	0.13	-0.14
	CH4@Sr	0.26	-0.03
	H4C@O	0.16	-0.14
	H4C@Sr	0.22	-0.07
(001) MnO_2 termination	CH4@O	-0.02	-0.18
	CH4@Mn	-0.11	-0.25
	H4C@O	0.14	-0.03
	H4C@Mn	0.09	-0.14
(110)	CH4@O	0.23	-0.03
	H4C@O	0.38	0.1

Note: CH_4@X and H_4C@X denote a CH_4 molecule adsorbed on X with one H pointing towards the surface and one where CH_4 was rotated by 180°, respectively.

Supplementary References

1 Petkovich, N. D., Rudisill, S. G., Wilson, B. E., Mukherjee, A. & Stein, A. Control of TiO2 Grain Size and Positioning in Three-Dimensionally Ordered Macroporous TiO2/C Composite Anodes for Lithium Ion Batteries. *Inorg. Chem.* **53**, 1100-1112, (2014).

2 van de Walle, A. *et al.* Efficient stochastic generation of special quasirandom structures. *Calphad* **42**, 13-18, (2013).

3 Henkelman, G., Uberuaga, B. P. & Jónsson, H. A climbing image nudged elastic band method for finding saddle points and minimum energy paths. *The Journal of Chemical Physics* **113**, 9901-9904, (2000).

4 Ciobîcă, I. M., Frechard, F., van Santen, R. A., Kleyn, A. W. & Hafner, J. A DFT Study of Transition States for C−H Activation on the Ru(0001) Surface. *The Journal of Physical Chemistry B* **104**, 3364-3369, (2000).

5 Zhang, C. J. & Hu, P. Methane transformation to carbon and hydrogen on Pd(100): Pathways and energetics from density functional theory calculations. *The Journal of Chemical Physics* **116**, 322-327, (2002).

6 An, W., Zeng, X. C. & Turner, C. H. First-principles study of methane dehydrogenation on a bimetallic Cu/Ni(111) surface. *The Journal of Chemical Physics* **131**, 174702, (2009).

7 Cheng, Z. *et al.* Oxygen vacancy promoted methane partial oxidation over iron oxide oxygen carriers in the chemical looping process. *Physical Chemistry Chemical Physics* **18**, 32418-32428, (2016).

8 Liu, Y. *et al.* In situ poly(methyl methacrylate)-templating generation and excellent catalytic performance of MnOx/3DOM LaMnO3 for the combustion of toluene and methanol. *Appl. Catal., B* **140–141**, 493-505, (2013).

9 Szabo, V., Bassir, M., Van Neste, A. & Kaliaguine, S. Perovskite-type oxides synthesized by reactive grinding: Part IV. Catalytic properties of LaCo1−xFexO3 in methane oxidation. *Appl. Catal., B* **43**, 81-92, (2003).

10 Belessi, V. C., Ladavos, A. K. & Pomonis, P. J. Methane combustion on La□Sr□Ce□Fe□O mixed oxides: bifunctional synergistic action of SrFeO3−x and CeOx phases. *Appl. Catal., B* **31**, 183-194, (2001).

11 Ren, Z. *et al.* Monolithically Integrated Spinel MxCo3−xO4 (M=Co, Ni, Zn) Nanoarray Catalysts: Scalable Synthesis and Cation Manipulation for Tunable Low-Temperature CH4 and CO Oxidation. *Angewandte Chemie International Edition* **53**, 7223-7227, (2014).

12 Xu, J. *et al.* Operando Raman Spectroscopy for Determining the Active Phase in One-Dimensional Mn1−xCexO2±y Nanorod Catalysts during Methane Combustion. *The Journal of Physical Chemistry Letters* **1**, 1648-1654, (2010).
